# Identification of major allergens in spices by mass spectrometry

**DOI:** 10.1186/2045-7022-3-S3-P55

**Published:** 2013-07-25

**Authors:** M Hummel, J Brockmeyer

**Affiliations:** 1 Institute of Food Chemistry, WWU Muenster, Muenster, Germany

## Background

About 3-4 % of the population in industrial countries are adversely affected by food allergens [1]. Spice allergy represents 2 % of these cases. Spices are one of the most attractive ingredients to add a unique taste to food and are often used in blends. Spice blends in general consist of a variety of potentially allergenic ingredients which complicates the identification of the particular offending component. Furthermore, such compositions may vary depending on local availability of ingredients and on manufacturer´s recipes. An increasing use of spices in food and cosmetics is observed in the last few decades. As is no curative therapy against food allergies has been established, consequent avoidance of allergens is mandatory for allergic individuals and precise labeling of prepacked food is necessary. Since 2005 the labeling of the 14 most common allergens (e.g. mustard, sesame and celery) is required by law [2].

## Methods

Sesame seeds, mustard seeds, celery root and celery stalk were purchased from a local supermarket and milled to a fine powder. After extraction, the protein mixture was stepwise separated by several analytical techniques (e.g. anion exchange chromatography, cation exchange chromatography, size exclusion chromatography). Additionally, selected target proteins were identified by HRMS (high resolution mass spectrometry).

## Results

Important proteinogenic allergens from sesame seeds and mustard seeds could successfully be purified und characterized. In the next step, this method will be applied to food samples with complex matrices.

## Conclusion

A precise and reliable diagnosis on component resolved level is the key to determine the appropriate therapeutic measures and to avoid unnecessary medical investigations. The intention of this project is to develop a method to detect smallest traces of allergens in food by using HRMS. Due to the ubiquitous presence of spices in food the purification and characterization of major spice allergens is important for allergen-related medical application.

## Disclosure of interest

None declared.
